# Single-cell transcriptomic profiling of bronchial lymph nodes reveals mechanisms of PRRSV escape from host adaptive immunity

**DOI:** 10.3389/fimmu.2026.1869357

**Published:** 2026-07-15

**Authors:** Shu-yuan Guo, Xuan Wang, Jiaying Zhu, Chang Liu, Hao Zhou, Yaqi Chen, Yu Bai, Haoran Liu, Wen-hai Feng

**Affiliations:** 1 Frontiers Science Center for Molecular Design Breeding, Beijing, China; 2 State Key Laboratory of Animal Biotech Breeding, Beijing, China; 3 Ministry of Agriculture Key Laboratory of Soil Microbiology, Beijing, China; 4 Department of Microbiology and Immunology, College of Biological Sciences, China Agricultural University, Beijing, China

**Keywords:** adaptive immunity, immune Evasion, PRRSV, RAG1^+^ CD4^+^ CD8^+^ DP T cells, single-cell RNA sequencing

## Abstract

**Introduction:**

Porcine reproductive and respiratory syndrome (PRRS), caused by porcine reproductive and respiratory syndrome virus (PRRSV), is characterized by impaired adaptive immune responses, including persistent viremia, delayed neutralizing antibody (nAb) development, and defective T-cell responses. However, the mechanisms by which PRRSV subverts adaptive immunity remain incompletely understood.

**Methods:**

We performed single-cell RNA sequencing of bronchial lymph nodes (BLNs) collected from piglets infected with the highly pathogenic PRRSV (HP-PRRSV) wild-type isolate HV or its attenuated derivative N29 to characterize immune cell composition, transcriptional programs, and intercellular communication associated with PRRSV infection.

**Results:**

HP-PRRSV HV infection reduced the proportions of multiple T-cell subsets, including naive T cells, proliferating T cells, and central memory T cells (Tcm), while increasing the proportion of B cells in BLNs. The infection also appeared to disrupt T-cell egress, potentially resulting in the accumulation of cytotoxic T lymphocytes (CTLs) within BLNs. In addition, HP-PRRSV HV impaired the crosstalk between T follicular helper cells and germinal center B cells, a key interaction required for antibody maturation, thereby potentially suppressing nAb production. Notably, we identified a previously unrecognized PRRSV-associated RAG1^+^ CD4^+^CD8^+^ double-positive (DP) T-cell subset with potential roles in lipid antigen recognition.

**Discussion:**

These findings suggest a multifaceted understanding of the PRRSV immune evasion mechanisms and may provide critical clues for vaccine design and antiviral strategies.

## Introduction

Porcine reproductive and respiratory syndrome (PRRS) is one of the most economically devastating diseases to the global swine industry (1). The causative agent, PRRS virus (PRRSV), is an enveloped single-stranded RNA virus belonging to the genus *Arterivirus*, leading to reproductive failures in sows and respiratory distresses in pigs of all ages (2). Globally, PRRSV is divided into two major genotypes: PRRSV-1 (European type) and PRRSV-2 (North American type) (3). In China, PRRS was first reported in 1995. Currently, PRRSV-2 is the dominant genotype circulating in China, with three subtypes being the most prevalent: highly pathogenic PRRSV (HP-PRRSV), NADC30-like PRRSV, and NADC34-like PRRSV (4, 5). Currently, there is no broad-spectrum, highly effective, and safe vaccine available for PRRSV (6).

PRRSV infection induces severe immunosuppression, with distinct disruptions to innate and adaptive immunity that collectively enable viral evasions (2). In innate immunity, the virus potently inhibits the production of type I interferon (IFN-I)—a central inducer of the antiviral state and a critical bridge linking innate and adaptive immunity (7–9). IFN-I is essential for the maturation of dendritic cells (DCs), their antigen-presenting function, and the efficient activation of T cells (10). Consequently, PRRSV-mediated suppression of the IFN pathway directly impairs antigen-presenting cell function, fundamentally undermining the initiation of subsequent adaptive immune responses.

In the context of adaptive immunity, PRRSV infection leads to remarkably delayed and deficient neutralizing antibody production (11). Within approximately one week post-infection, the host generates virus-specific antibodies; however, these early immunoglobulins are predominantly non-neutralizing (12, 13). Rather than conferring protection, they may promote infection through antibody-dependent enhancement (ADE), whereby the Fc region of antibodies binds to Fcγ receptors on PAMs to facilitate PRRSV infection (14, 15). Truly protective neutralizing antibodies typically do not emerge until around four weeks post-infection, creating an extended window for PRRSV replication and persistence (13). Cellular immune responses are similarly impaired (16, 17). PRRSV-specific T cell activation is both delayed and weak. Critically, cytotoxic T lymphocytes (CTLs) remain undetectable until 3–4 weeks post-infection and display limited functional capacity, thereby failing to effectively clear infected cells (2). This defective T cell response is closely associated with PRRSV-induced impairment of DC function (18). Furthermore, PRRSV infection can cause severe thymic atrophy (19), disrupting T cell development and output, and thereby exerting a long-term negative impact on the adaptive immune system.

While previous studies have described the manifestations of PRRSV-induced adaptive immunosuppression, the underlying mechanisms remain poorly defined. Recent single-cell transcriptomic studies have characterized the alveolar immune landscape (20) and detailed the immune response dynamics of alveolar macrophages, the primary target cells of PRRSV, which significantly advances our understanding of the innate immune response to PRRSV (21, 22). These findings primarily focus on the site of initial infection and innate immunity. Moreover, current understanding of the adaptive immunosuppression still largely derives from analyses of peripheral blood or *in vitro* models, which lack the resolution to systematically dissect the complex cellular interactions within the immunological microenvironment. Given that the lung is the primary site of PRRSV infection and the bronchial lymph nodes (BLNs) are its draining lymphoid organs, we employ single-cell RNA sequencing (scRNA-seq) on BLNs to elucidate the strategies by which PRRSV disrupts adaptive antiviral immunity.

In this study, we performed a comparative single-cell transcriptomic analysis of BLNs from pigs infected with either a HP-PRRSV wild-type (WT) isolate (HV) or its attenuated counterpart (N29), which was generated by codon pair deoptimization of the nsp2 and nsp9 gene of HV. As previously reported, N29 exhibits attenuated pathogenicity while effectively inducing neutralizing antibody production and CD8^+^ T cell responses (23). This experimental design allows for a direct comparison of the immune landscapes under conditions of pathogenic infection versus protective immunization, thereby facilitating the identification of precise viral evasion mechanisms. Our analysis revealed that, unlike N29 infection, HP-PRRSV wild-type HV infection was associated with remodeling of the immune landscape of BLNs, including altered cellular composition and potentially disrupted intercellular crosstalk. A key finding was the apparent impairment of crosstalk between T follicular helper (Tfh) cells and germinal center B cells in the light zone, which may contribute to the compromised neutralizing antibody response. Moreover, we discovered a novel virus-associated T cell subset defined by the co-expression of *RAG1, CD4*, and *CD8*. Collectively, our findings offer potential insights into the mechanisms underlying PRRSV-mediated adaptive immunosuppression.

## Materials and methods

### Cells and viruses

Porcine alveolar macrophages (PAMs) were obtained from lung lavage of 6–8-week-old specific pathogen-free (SPF) piglets (the Large White breed). PAMs were cultured in RPMI 1640 (Gibco, NE, USA) with 10% FBS (Gibco, NE, USA), 1% penicillin and streptomycin. Marc-145 cells (ATCC CRL-12231) were cultured in Dulbecco modified Eagle medium (DMEM; Gibco, NE, USA) supplemented with 10% FBS, and 1% penicillin and streptomycin. All cells were cultured at 37 °C in an incubator with 5% CO2.

The HP-PRRSV strain HV was propagated and titrated in porcine alveolar macrophages (PAMs). The attenuated N29 strain—constructed by our laboratory and derived from HV—was propagated and titrated in Marc-145 cells. Viral supernatants were collected from the respective cell cultures, and the 50% tissue culture infective dose (TCID_50_) of each virus was determined using the Reed–Muench method.

### Experimental animals and infection

Four-week-old healthy conventional Large White–Dutch Landrace crossbred piglets (n = 9) sourced from a PRRSV-negative farm were used in the experiment. All pigs were further confirmed to be free of PRRSV antibodies by using commercial enzyme-linked immunosorbent assay (ELISA) kits (IDEXX Lab. and Ingenasa). PRRSV, classical swine fever virus (CSFV), porcine circovirus 2 (PCV2), and pseudorabies virus (PRV) were also confirmed to be free using PCR or RT-PCR. These piglets were divided into three groups (n = 3 per group): mock-infected, HV-infected, and N29-infected, with female and male animals randomly assigned to each group. On day 0, piglets in the HV and N29 groups were intranasally inoculated with 2 mL of the respective PRRSV strain at a titer of 1 × 10^5^ TCID_50_/mL. Mock-infected piglets received an equivalent volume of 0.9% NaCl. All animals were housed under standard conditions with ad libitum access to feed and water. Clinical signs including cough, dyspnea, anorexia, diarrhea, lameness, and tremor were monitored daily. Rectal temperature was measured every other day post-inoculation. Serum samples were collected at 0, 4, 7, and 10 dpi for viral titer determination. All experimental procedures involving animals were approved by the Ethical Review Committee for Laboratory Animal Welfare and Animal Experimentation of China Agricultural University (Approval no. AW42115202-3-1), and strictly followed the relevant regulations and guidelines of the committee.

### Sample collection

At 10 dpi, all piglets were euthanized by intravenous injection of sodium pentobarbital (100 mg/kg) according to approved ethical guidelines. Tissue samples were collected for subsequent analysis. BLN samples were processed for single-cell RNA sequencing, while lung and other tissues were divided for viral load quantification and histopathological examination.

### RNA extraction from tissues and serum

Total RNA was extracted from 10 tissues (including BLNs, spleen, lung, etc.) and serum samples using standardized protocols. For tissue samples, approximately 50 mg of each tissue was thoroughly homogenized in TRIzol reagent (CW Biotech, Beijing, China) using a tissue homogenizer, followed by the addition of chloroform to induce phase separation. For serum samples, 200 μL of each was first centrifuged to remove cellular debris, then mixed directly with TRIzol reagent and processed in the same manner as the tissue samples. In both cases, the aqueous phase containing RNA was collected, and RNA was precipitated with isopropanol, washed with 75% ethanol, air-dried, and finally re-suspended in RNase-free water. RNA concentration and purity were assessed using a NanoDrop 2000 spectrophotometer (A260/A280 ratio 1.8–2.0). All RNA samples were stored at -80 °C until use.

### Real-time RT-PCR

cDNA synthesis was performed using 100 ng of RNA (isolated from serum or tissues) as the template, with the HiFiScript cDNA Synthesis Kit (Meiwei Biotechnology Co., Ltd.). Real-time PCR was conducted on an ABI ViiA7 Real-Time PCR System. Specific primers targeting the N protein gene of PRRSV were used, with the sequences as follows: forward primer: 5’-ACAACGGCAAGCAGCAAAAG-3’; reverse primer: 5’-CTGGACTGGTTTTGTTGGGC-3’. The reaction was carried out using the SYBR Green Real-Time PCR Master Mix (Meiwei Biotechnology Co., Ltd.), strictly following the manufacturer’s instructions. A standard curve was generated using serial dilutions of a PRRSV standard, where the viral titer of the dilutions ranged from 10^0^ to 10^7^ TCID_50_/mL.

### Serum neutralization assay

Test sera were first heat-inactivated in a 56 °C water bath for 1 hour. Subsequently, the inactivated sera were subjected to 2-fold serial dilution using RPMI 1640 medium. Each diluted serum sample was then mixed with an equal volume of HV virus suspension (200 TCID_50_/0.1 mL), followed by incubation at 37 °C for 60 minutes to allow antibody-virus binding. Thereafter, the serum-virus mixture was added to PAMs cultured in 96-well plates. At 36 hours post-inoculation, viral infection was assessed by immunofluorescence assay. A 50% reduction in viral load relative to the virus-only control (i.e., virus mixed with RPMI 1640) was defined as effective neutralization. Neutralizing antibody titers were expressed as log_2_ values.

### Immunofluorescence assay

Following 36 hours of co-culture with the serum-virus mixture, PAMs were subjected to immunofluorescence staining. Briefly, the cells were fixed with cold 4% paraformaldehyde for 10 min, and then blocked with 5% goat serum for 30 min at room temperature. Subsequently, the cells were incubated with anti-PRRSV N protein monoclonal antibody at 37 °C for 1 h, followed by three washes with PBS. Next, an Alexa Fluor™ 594-conjugated goat anti-mouse IgG secondary antibody was applied and incubated at 37 °C for 1 h in the dark. After final washes, the cells were examined using a fluorescence microscope.

### HE staining for tissue sections

To clarify the pathological damage of porcine respiratory and lymphatic tissues caused by PRRSV infection, tissue section preparation and HE staining were performed to observe microscopic morphological changes. Tissues (lung, BLNs, and thymus, etc.) were fixed in 4% paraformaldehyde for 24–48 hours. They were then dehydrated through gradient ethanol, cleared in xylene, and embedded in paraffin. Paraffin blocks were sectioned into 4–5 μm slices, which were dewaxed in xylene and rehydrated via gradient ethanol. For staining, sections were incubated in Harris hematoxylin for 5–10 minutes to label cell nuclei, followed by differentiation in 1% hydrochloric acid-ethanol and bluing in running water. They were then stained with 0.5% eosin for 1–3 minutes to label cytoplasm, dehydrated in ethanol, cleared in xylene, and mounted with neutral balsam. Stained sections were observed under a light microscope to assess tissue pathological changes.

### Single-cell suspension preparation

Lymph node tissues were cut into approximately 0.5-mm^3^ pieces in the RPMI-1640 medium (Invitrogen) with 1% Penicillin/Streptomycin, and enzymatically digested with the MACS Tumor Dissociation Kit mouse (Miltenyi Biotec) at 37 °C for 30 min with agitation according to the manufacturer’s instructions. The dissociated cells were subsequently passed through a 70 µm and 40 µm cell-strainer (BD) and centrifuged at 300×g for 10 min. After the supernatant was removed, the pelleted cells were suspended in red blood cell lysis buffer (Thermo Fisher) and incubated on ice for 2 min to lyse red blood cells. After washing twice with PBS (Invitrogen), the cell pellets were re-suspended in PBS (containing 0.04% BSA).

### Single-cell RNA-seq library construction and sequencing

Single-cell RNA-seq libraries were prepared using the DNBelab C Series High-throughput Single-Cell RNA Library Preparation Set (MGI, #940-000519-00). Following the manufacturer’s protocol, single-cell suspensions were subjected to droplet encapsulation, reverse transcription, and cDNA amplification. The resulting cDNA was then fragmented into 300–500 bp segments for the construction of indexed sequencing libraries. Library quality was assessed using the Qubit ssDNA Assay Kit (Thermo Fisher Scientific) and the Agilent Bioanalyzer 2100. Final libraries were sequenced on a DNBSEQ-T7 platform with a paired-end strategy, generating 30-bp read 1 (containing cell barcodes and UMIs), 100-bp read 2 (for transcript sequences), and a 10-bp sample index read.

### Single-cell RNA sequencing data processing

Sequencing data were processed using the DNBelab C Series scRNA-analysis pipeline (v2.1.3, https://github.com/MGI-tech-bioinformatics/DNBelab_C_Series_scRNA-analysis-software). Raw sequencing reads were demultiplexed based on sample indices, followed by cell barcode assignment and UMI counting using the pipeline’s default parameters. Processed reads were aligned to the Sus scrofa reference genome (Ensembl V111) using STAR (v2.7.2b). Valid cells were identified based on UMI count distributions using the “barcodeRanks (x = umi_counts)” function from the DropletUtils package (v1.8.0). Cells with UMI counts below the automatically determined threshold were considered background droplets and removed. Additional quality control metrics were applied, including exclusion of cells with fewer than 500 detected genes and cells with mitochondrial gene content exceeding 25%. Doublets were identified and removed using DoubletFinder (v2.0.3). Following quality control, gene expression matrices from all samples were integrated using the Harmony algorithm (v1.1.0) to correct for batch effects. Data normalization was performed using SCTransform (v0.3.5) with regression of mitochondrial percentage and cell cycle scores. Highly variable genes were selected for downstream analysis using the “vst” selection method. The downstream single-cell data analysis was performed using the OmicStudio tools created by LC-BIO Co., Ltd (Hangzhou, China) at https://www.omicstudio.cn/cell.

### Cell clustering and annotation

Following quality control, we performed principal component analysis on the normalized gene expression matrix. Graph-based clustering was applied using the top 20 principal components, with cluster resolution set to 0.8. Cell clusters were visualized using UMAP. Cell types were annotated based on established marker genes.

### Subpopulation re-clustering

For deeper analysis of T or B cell heterogeneity, we extracted all T or B cells and performed sub-clustering at higher resolution (1.2), identifying subsets based on specific markers (**Supplementary Table 1**).

### Differential gene expression analysis

Differentially expressed genes (DEGs) between experimental groups were identified using the Wilcoxon rank sum test with a threshold of |log2(fold change)| > 0.26 and adjusted *p-value < 0.05*. DEGs were visualized using volcano plots and heatmaps.

### Cell-cell communication analysis

CellPhoneDB (v5.0.0) was employed to infer and analyze intercellular communication networks. The tool was applied to identify significantly altered ligand-receptor interactions between HV- and N29-infected groups, with interactions visualized using circle plots and heatmaps.

### Pseudotime trajectory analysis

Monocle2 (v2.22.0) was used to construct single-cell trajectories and order cells along pseudotime. Cells were ordered based on gene expression patterns to reconstruct developmental pathways, with branch points analyzed to identify genes associated with cell fate decisions.

### Functional enrichment analysis

Gene Ontology (GO) and KEGG pathway enrichment analyses were performed using the clusterProfiler package (v4.2.2). DEGs were mapped to biological processes, molecular functions, and signaling pathways, with terms considered significant at an adjusted *p-value < 0.05*. Results were visualized using dot plots and bar graphs.

### Statistical considerations

Statistical analyses were performed using GraphPad Prism and R (v4.1.2). For wet-lab experiments, differences were analyzed using *Student’s t-test*. Significance is denoted in the figures as follows: *, *P* < 0.05; **, *P* < 0.01; ***, *P* < 0.001, and ns, not significant. In scRNA-seq data analysis, differential expression of every cluster was calculated by using the bimod test as implemented in Seurat FindMarkers function.

## Results

### Clinical outcomes of PRRSV infections with HP-PRRSV WT isolate HV and its attenuated N29

**Figure 1:**
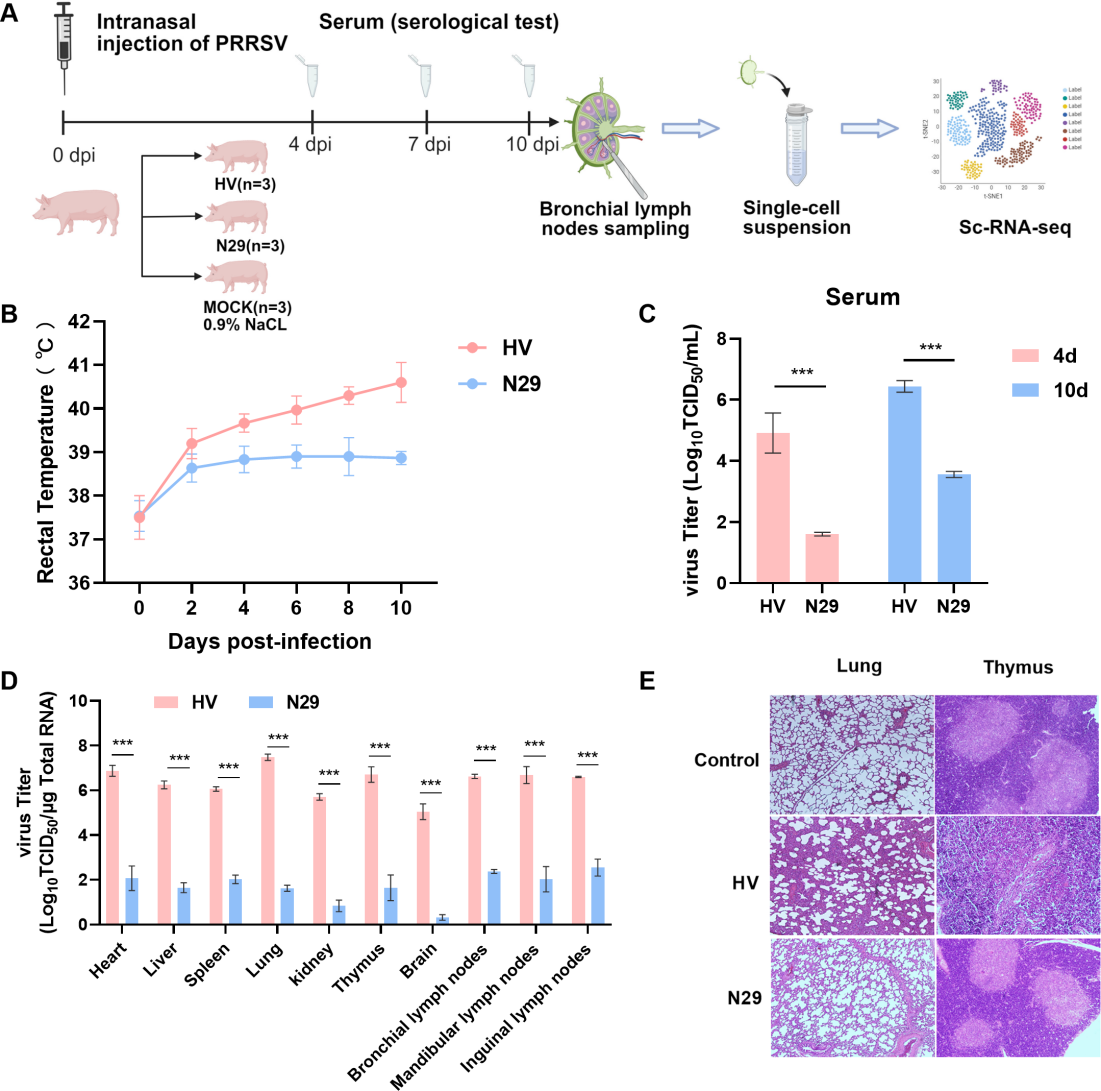
Clinical outcomes of PRRSV infections with HP-PRRSV WT isolate HV and its attenuated N29. **(A)** Schematic overview of the experimental design. Figure created with BioRender (https://biorender.com). **(B)** Rectal temperature was measured every two days for HV- and N29- infected pigs after virus infection. **(C)** Analysis of viral load in serum samples from HV- and N29- infected pigs at indicated times post-infection. **(D)** Bar plot showing viral load analysis in tissues collected at necropsy (10 dpi) from HV- and N29-infected pigs. LN, lymph node. **(E)** Histological examination of lung and thymus tissues collected at necropsy (10 dpi) from MOCK, HV-, and N29-infected pigs, to evaluate PRRSV-induced tissue pathological changes.

To validate the pathogenic and virulence differences between HP-PRRSV WT isolate HV and its attenuated N29 in our experimental setting, we first characterized the clinical and virological outcomes of the infections. Four-week-old pigs (n = 3 per group) were intranasally inoculated with 2 ml of 1 × 10^5^ TCID_50_/mL of HV, N29, or mock-infected cell culture supernatant, and monitored for 10 days post-infection (dpi) (**Figure 1A**).

HV infection induced severe clinical manifestations, including sustained fever from 4 to 10 dpi (**Figure 1B**) and obvious signs of respiratory distresses. In contrast, N29-infected pigs exhibited no fever and minimal clinical signs as previously reported (23). Quantitative RT-PCR (qRT-PCR) results revealed that serum viral titers were significantly higher in HV-infected pigs compared to N29-infected pigs, with a consistent difference of approximately 10^3–^10^4^ TCID_50_/mL observed at both 4 and 10 dpi (**Figure 1C**). All pigs were euthanized at 10 dpi for tissue analysis. qRT-PCR assays showed that viral RNA loads in N29-infected pigs were significantly lower across multiple tissues (e.g. lungs, BLNs, and brain) (**Figure 1D**). Hematoxylin-eosin (HE) staining further demonstrated that HV infection induced severe pathological lesions, such as marked interstitial pneumonia and thymic atrophy, whereas no obvious tissue damages were observed in N29-infected pigs (**Figure 1E**). Together, these results confirm that HV is highly pathogenic in pigs, while N29 retains minimal virulence, thereby validating the suitability of our experimental system for subsequent mechanistic investigations.

### Cellular composition of bronchial lymph nodes following infection with HP-PRRSV strains of different virulence

**Figure 2:**
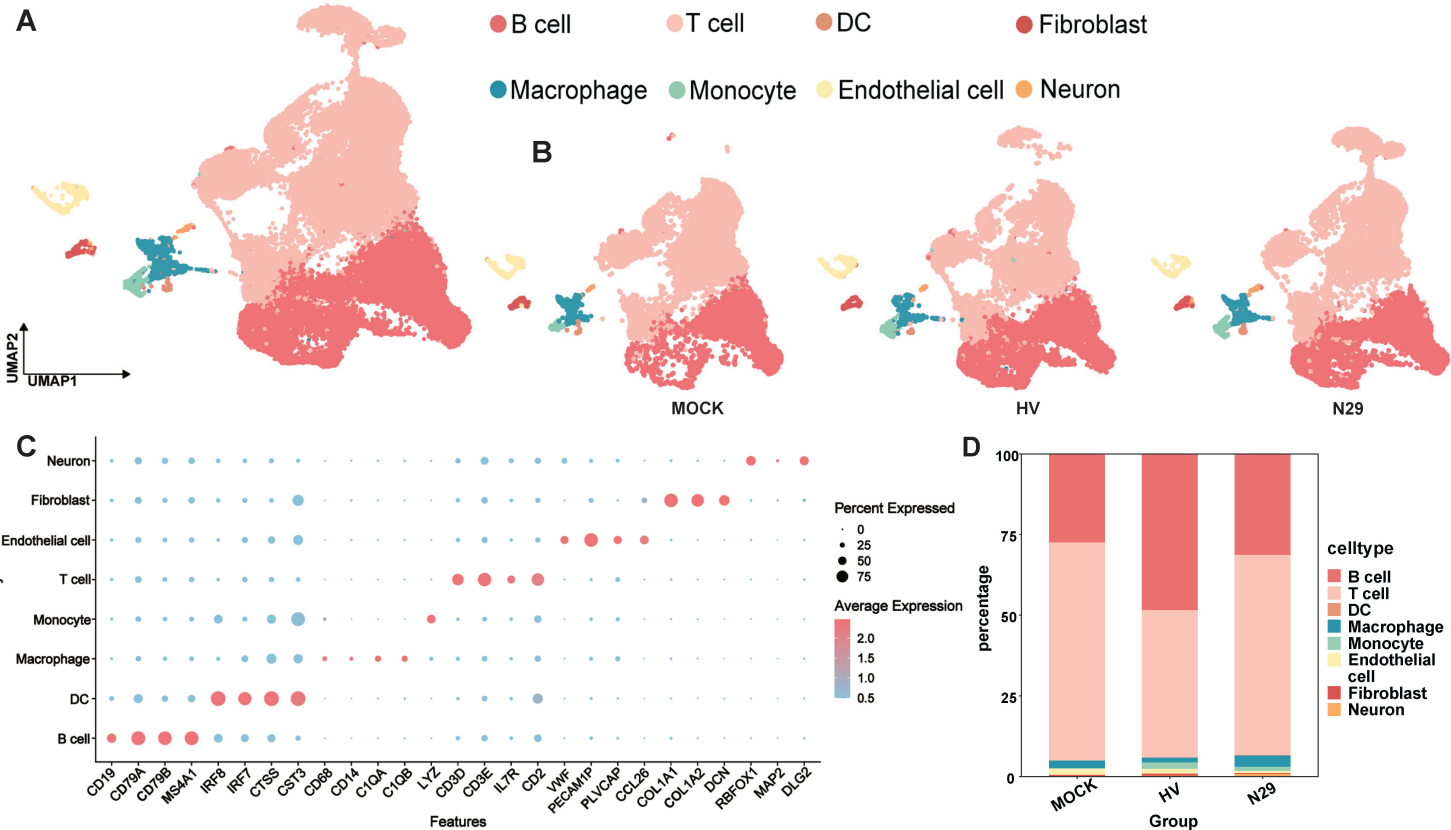
Cellular composition of bronchial lymph nodes following infection with HP-PRRSV strains of different virulence. **(A)** Uniform Manifold Approximation and Projection (UMAP) plot showing 65,582 cells isolated from BLNs of pigs in the MOCK control (n = 3), HV-infected (n = 3), and N29-infected (n = 3) groups. A total of 22,525, 22,416, and 19,387 cells were recovered from the MOCK, HV, and N29 groups, respectively. Cells are colored by annotated cell type. **(B)** UMAP plot of all 65,582 BLN cells, colored by experimental group (MOCK, HV, N29) to visualize inter-group cell distribution. **(C)** Dot plot displaying the expression of key marker genes used for cell type annotation. Each dot represents the average normalized expression (color intensity) and the percentage of cells expressing the gene (dot size) within a given cell subset. **(D)** Stacked bar plot quantifying the relative proportion of immune cell clusters in BLNs from HV- and N29-infected pigs. Proportions were normalized to the total number of immune cells in each sample to reflect cluster abundance changes post-infection.

To clarify how PRRSV infection alters the cellular makeup of lymphoid tissues involved in pulmonary immune responses, we performed scRNA-seq on BLN cells collected at 10 days post-infection (**Figure 1A**). After quality control, a total of 65,582 high-quality cells were obtained from 9 BLN samples. Unsupervised clustering followed by uniform manifold approximation and projection (UMAP) visualization identified eight major cell population (**Figures 2A, B**), which were annotated according to well-established marker genes (**Figure 2C**). Overall, T cells (*CD3D*, *CD3E*) and B cells (*CD79A*, *MS4A1*) constituted the vast majority (> 90%) of the cells, while myeloid cells—including macrophages (*C1QA*, *C1QB*) and dendritic cells (*IRF8*, *IRF7*)—comprised a relatively minor fraction (**Figure 2D**).

Comparative analysis across infection groups revealed distinct alterations in immune cell proportions relative to mock-infected controls (**Figure 2D**). HV infection induced a marked expansion of B cells (48.41% vs. 28.01% in mock, ~1.7-fold increase) and a concomitant reduction in T cell frequency (45.62% vs. 66.93% in mock, ~0.68-fold relative to mock). Additionally, we observed non-significant trends toward a moderate decrease in macrophage proportion (1.5% vs. 2.41% in mock).

In contrast, the cellular composition in N29-infected pigs closely resembled that of the mock group, with only minimal deviations. Direct comparison between N29 and HV groups revealed an approximately 1.6-fold lower B cell proportion (30.68% vs. 48.41%) and an approximately 1.4-fold higher T cell proportion (62.46% vs. 45.62%) in N29-infected pigs (**Figure 2D**). The consistent trends suggest that the attenuated N29 strain causes substantially less disruption of BLN immune homeostasis than the wild-type HV strain. This differential impact on immune cell architecture may underlie the distinct capacities of the two viral strains to elicit adaptive immunity.

### HV infection reduces naive, proliferating, and central memory T cell population relative to N29

**Figure 3:**
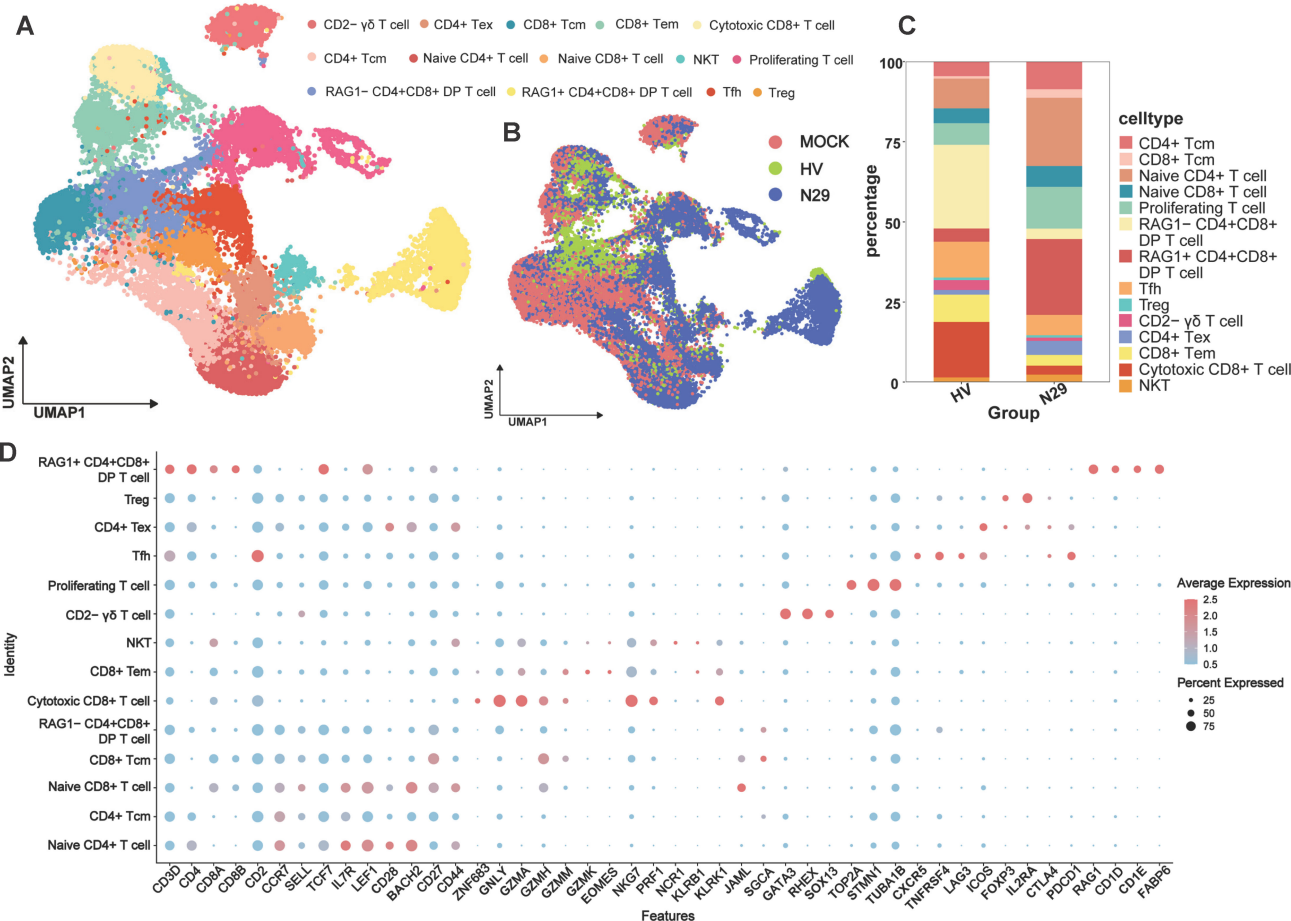
HV infection reduces naive, proliferating, and central memory T cell population relative to N29. **(A)** UMAP visualization of T lymphocyte subsets generated by re-clustering all T cells. Cells are colored by 14 annotated T cell subsets. **(B)** UMAP plot of re-clustered T cells, colored by experimental group (MOCK, HV, N29). **(C)** Dot plot displaying the expression of key marker genes for each of the 14 T cell subsets. Dot color intensity represents the average normalized expression of the gene, and dot size indicates the percentage of cells expressing the gene within the subset. **(D)** Stacked bar plot quantifying the relative proportion of each T cell subset in HV- and N29-infected pigs. Proportions were normalized to the total number of T cells in each sample to reflect subset abundance changes between the two infected groups.

To dissect the specific alterations in T cell subsets underlying the overall reduction in T cell proportion upon HV infection, we performed a focused re-clustering and subpopulation analysis of T lymphocytes. We extracted all T cells (annotated by CD3E expression in the global cell atlas) for unsupervised re-clustering, which successfully yielded 14 distinct T cell subsets (**Figures 3A, B**). These subsets were annotated based on canonical marker genes and functional signatures (**Figure 3C**).

The naive T cell pool, which serves as the primary reservoir for viral antigen recognition, was significantly reduced in HV-infected pigs compared to that in the N29 group. Specifically, the proportions of CD4^+^ naive T cells (22.03% in HV vs. 58.04% in N29) and CD8^+^ naive T cells (27.69% in HV vs. 44.81% in N29) were significantly lower (**Figure 3D**). These findings suggest that HV infection depletes the host’s naive T cell repertoire, potentially affecting its capacity to generate *de novo* adaptive immunity against the virus. Beyond naive T cells, proliferating T cells—critical for clonal expansion of antiviral effectors—were ~2.2-fold lower in HV-infected BLNs (24.95% in HV vs. 54.87% in N29, **Figure 3D**), implying that HV might suppress T cell population expansion. Central memory T cells (Tcm) (24), essential for sustaining long-term immunity, were also affected: both CD4^+^ Tcm (18.15% in HV vs. 39.28% in N29) and CD8^+^ Tcm (3.14% in HV vs. 12.73% in N29) frequencies were significantly reduced (**Figure 3D**), indicating a compromised establishment of durable immunological memory following HV infection. In summary, HV infection led to a coordinated decline in naive, proliferating, and central memory T cells within BLNs compared to N29 infection. These broad differences across multiple T cell compartments likely underlie the defective cellular immunity observed during HP-PRRSV infection.

### HV infection may inhibit the egress of cytotoxic T lymphocytes from bronchial lymph nodes

**Figure 4:**
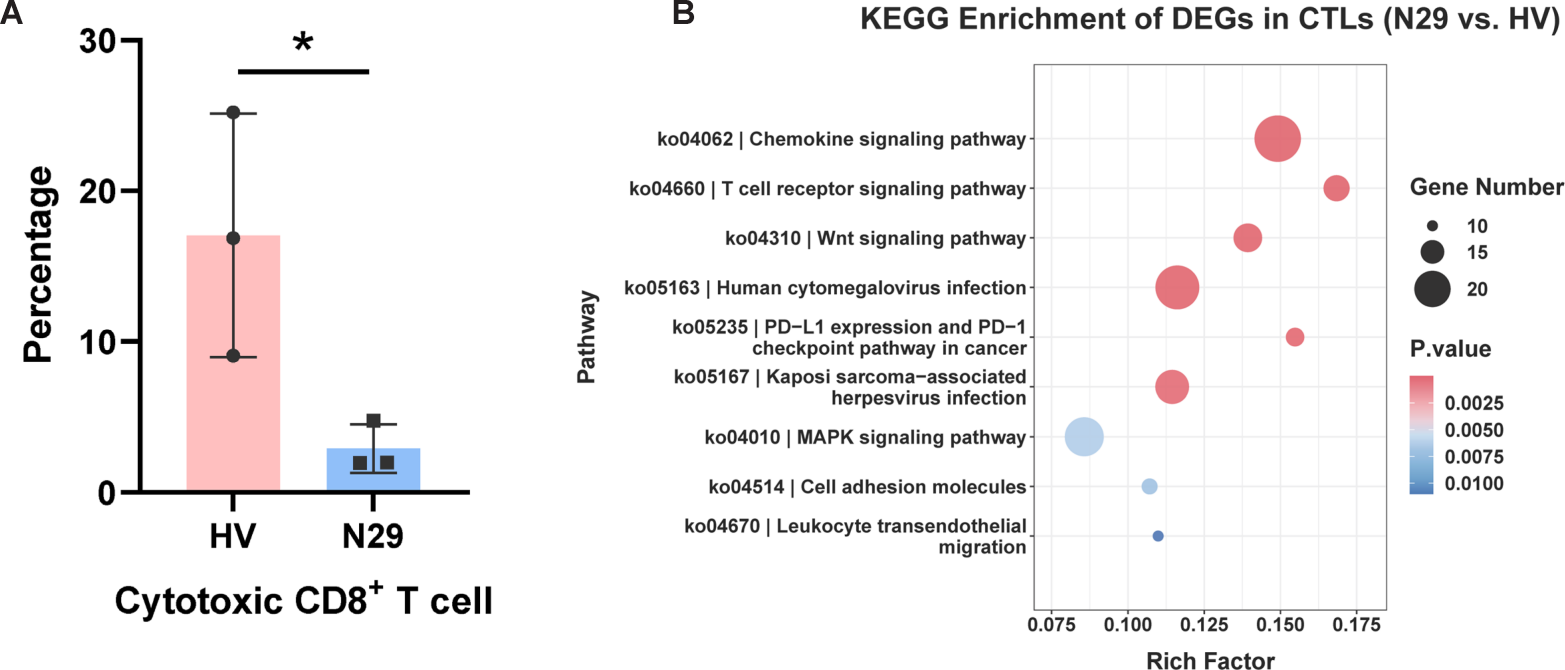
HV infection may inhibit the egress of cytotoxic T lymphocytes from bronchial lymph nodes. **(A)** Bar plot showing the relative percentage of cytotoxic T lymphocytes (CTLs) among total T cells in pigs infected with HV vs. N29. **(B)** Kyoto Encyclopedia of Genes and Genomes (KEGG) enrichment analysis of DEGs in CTLs between HV- and N29-infected pigs.

Despite an overall reduction in total T cells, the proportion of cytotoxic T lymphocytes (CTLs) was paradoxically elevated in the BLNs of HV-infected pigs (**Figure 4A**). We hypothesize that this accumulation might result from a disruption in CTL emigration, rather than enhanced local proliferation. To explore the molecular basis of this phenotype, we compared the transcriptional profiles of CTLs from HV- and N29-infected BLNs.

KEGG enrichment analysis of differentially expressed genes (DEGs) between HV- and N29-derived CTLs revealed that pathways governing immune cell migration and signaling were significantly down-regulated in the HV group (**Figure 4B**). Keys among these were the *Chemokine signaling pathway* (ko04062), *Cell adhesion molecules* (ko04514), and *Leukocyte transendothelial migration* (ko04670)—all directly implicated in lymphocyte trafficking. Additionally, pathways such as the *T cell receptor signaling pathway* (ko04660) and *MAPK signaling pathway* (ko04010) were also enriched, suggesting broader alterations in T cell activation and motility. Compared to the N29 group, CTLs from HV-infected pigs exhibited dysregulated expression of genes within these critical pathways, likely impairing their ability to egress from BLNs and home to pulmonary sites of infection. This molecular dysregulation suggests a candidate mechanism for the observed accumulation of CTLs in the BLNs during HV infection.

### A novel RAG1^+^ CD4^+^ CD8^+^ DP T cell subset associated with PRRSV infection

**Figure 5:**
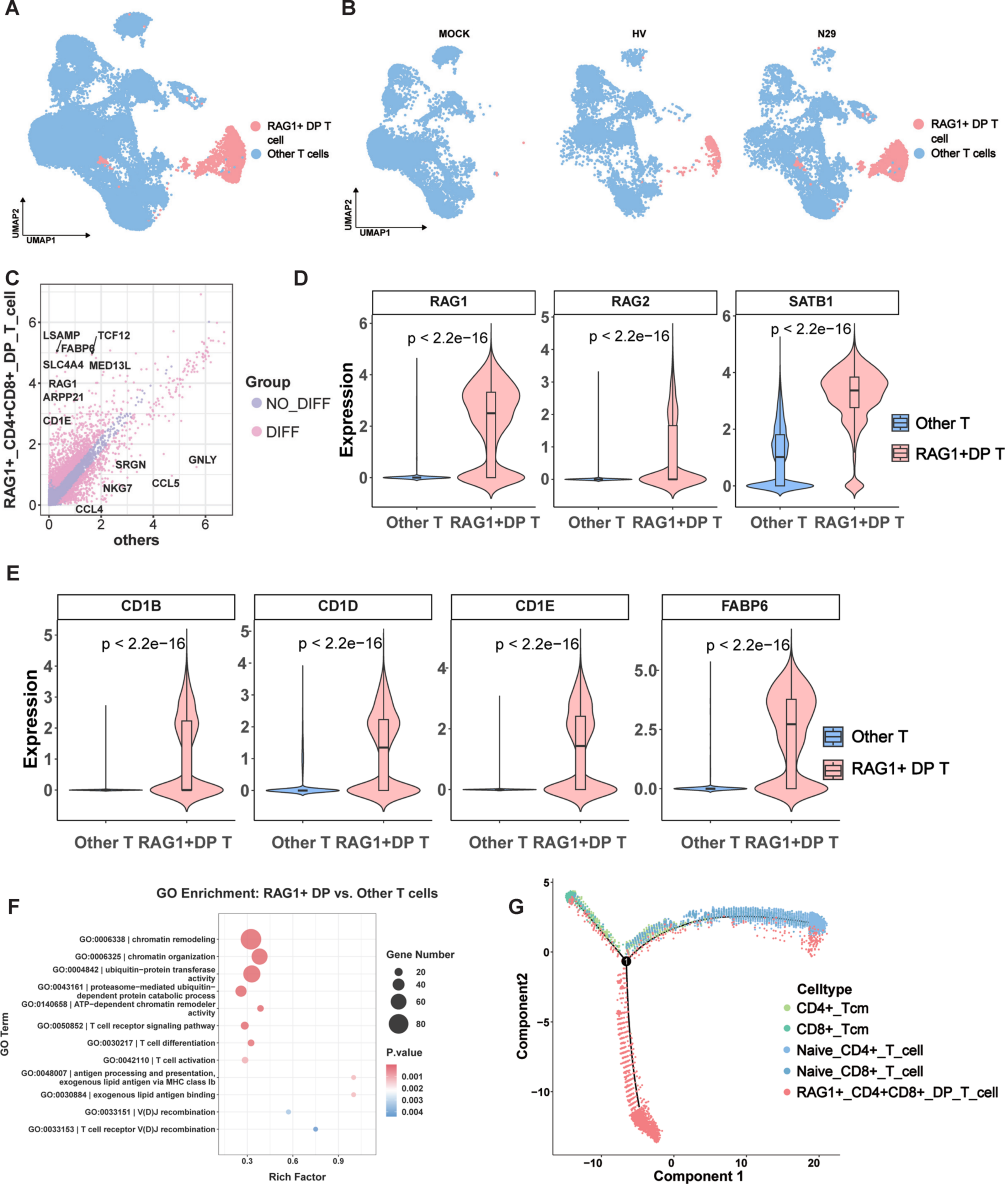
A novel RAG1^+^ CD4^+^ CD8^+^ DP T Cell Subset associated with PRRSV infection. **(A)** UMAP projection of single-cell transcriptomes from porcine BLNs, colored by cell type. The novel RAG1^+^ DP T cell subset is highlighted in deep pink. **(B)** UMAP of RAG1^+^ DP T cell and other T cells with group annotations (MOCK, HV, and N29). **(C)** Scatter plot identifying the top 10 upregulated and downregulated genes in the RAG1^+^ DP T cell subset compared to other T cells. **(D, E)** Violin plots depicting the expression of *RAG1, RAG2, SATB1* **(D)** and *CD1B, CD1D, CD1E, FABP6* **(E)** in RAG1^+^ DP T cells versus other T cell subsets. **(F)** Gene Ontology (GO) enrichment analysis of DEGs in RAG1^+^ DP T cells relative to other T cells. **(G)** Pseudotime trajectory analysis revealing the developmental relationship between the RAG1^+^ DP T cells, naive T cells, and Tcm.

During our single-cell transcriptomic analysis of BLNs, we identified a novel and unanticipated T cell subset defined by the co-expression of *RAG1* (recombination-activating gene 1), *CD4*, and *CD8* (**Figure 5A**). *RAG1* is conventionally restricted to developing lymphocytes in primary lymphoid organs such as the thymus, where it mediates V(D)J recombination to diversify immune cell receptor repertoire (25–28). Its expression is typically silenced in mature peripheral T cells, making the emergence of a RAG1^+^ CD4^+^ CD8^+^ double-positive (DP) population in peripheral lymphoid tissue particularly remarkable. Notably, this subset was observed only in PRRSV-infected pigs and was undetectable in mock-infected controls (**Figure 5B**), and the attenuated N29 strain triggered a higher proportion of RAG1^+^ CD4^+^ CD8^+^ DP T cells (hereafter referred to as RAG1^+^ DP T cell) compared to the highly pathogenic HV strain (**Figure 5B**), suggesting a possible role in the protective immune response.

To molecularly characterize this unconventional population, we performed differential gene expression analysis between RAG1^+^ DP T cells and all other T cell subsets. The top up-regulated genes included *RAG1*, *CD1E*, *FABP6*, *TCF12*, *ARPP21*, and *MED13L* (**Figure 5C**), which may point to involvement in lipid antigen presentation and T cell receptor (TCR) remodeling. Consistent with this, we observed significantly elevated expression of V(D)J recombination-related genes (29) (*RAG1*, *RAG2*, *SATB1*; **Figure 5D**) and lipid antigen presentation-associated genes (30) (*CD1B*, *CD1D*, *CD1E*, *FABP6*; **Figure 5E**) in this subset compared to other T cells. Gene ontology (GO) enrichment analysis also suggested a significant enrichment for biological processes including “V(D)J recombination,” “chromatin organization,” “T cell receptor signaling,” and “lipid antigen presentation via MHC class Ib” (**Figure 5F**). These molecular features collectively suggest that the RAG1^+^ DP T cells may undergo peripheral TCR re-editing and engage in unconventional lipid-driven immune recognition.

Additionally, pseudotime trajectory analysis placed this subset on a distinct developmental branch, clearly separated from the conventional naive-to-central memory T cell continuum (**Figure 5G**), implying that it might represent an independent lineage potentially arising from a PRRSV-triggered differentiation program rather than an intermediate state in conventional T cell maturation. Taken together, these results indicate that there might be a novel RAG1^+^ DP T cell subset associated with PRRSV infection in a virulence-dependent manner, which exhibits a unique transcriptional signature of peripheral TCR recombination and lipid antigen presentation capacity. Its preferential appearance in the N29-infected pigs suggests a possible role in protective immunity, offering new insights into PRRSV immunobiology.

### HV may impair humoral immunity by affecting Tfh function and Tfh-germinal center B cell crosstalk

**Figure 6:**
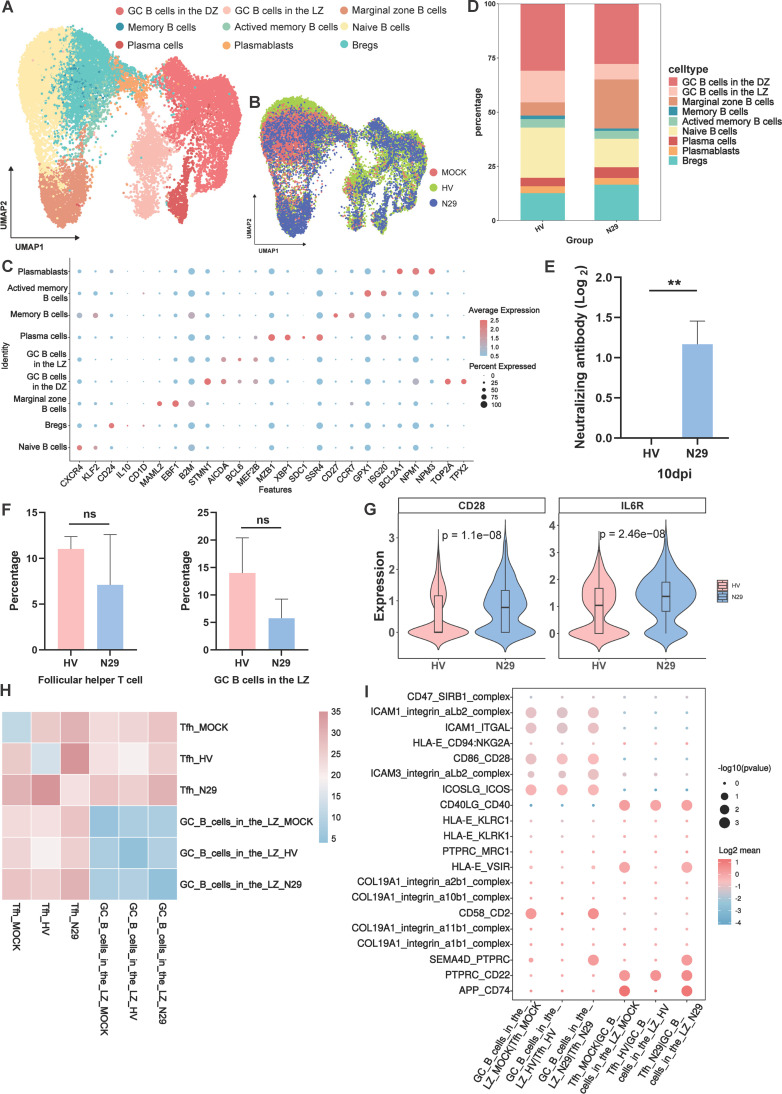
HV may impair humoral immunity by affecting Tfh function and Tfh-germinal center B Cell crosstalk. **(A)** UMAP visualization of B lymphocyte subsets generated by re-clustering all B cells. Cells are colored by 9 annotated B cell subsets. **(B)** UMAP plot of re-clustered B cells, colored by experimental group (MOCK, HV, N29). **(C)** Dot plot displaying the expression of key marker genes for each of the nine B cell subsets. Dot color intensity represents the average normalized expression of the gene, and dot size indicates the percentage of cells expressing the gene within the subset. **(D)** Stacked bar plot quantifying the relative proportion of each B cell subset in HV- and N29-infected pigs. Proportions were normalized to the total number of B cells in each sample to reflect subset abundance changes between the two infected groups. **(E)** Neutralizing antibody response at 10 d post-HV or nsp29 infection was determined by serum neutralization assay. Samples with no neutralization at the lowest dilution (1:1) are plotted at the assay cutoff (log_2_ titer = 0). **(F)** Bar plot showing the relative percentage of germinal center (GC) B cells in the light zone (among total B cells) and follicular helper T cells (Tfh) (among total T cells) in HV- vs. N29-infected pigs. **(G)** Violin plots depicting the expression of *CD28* and *IL6R* in Tfh cells between HV- and N29-infected groups. **(H)** Heatmap quantifying the differential cell-cell communication probability between Tfh cells and LZ GC B cells in HV- vs. N29-infected conditions. **(I)** Dot plot generated by CellPhoneDB, showing potential ligand-receptor pairs between Tfh cells and LZ GC B cells post HV or N29 infection. Dot color represents the mean expression of the ligand-receptor pair across the two cell subsets, and dot size is proportional to -log_10_ (P-value).

We next examined whether humoral immunity was similarly affected via dysregulation of the B cell compartment in BLNs. Unsupervised clustering of B cells identified nine distinct subpopulation (**Figures 6A, B**), such as naive B cells, germinal center B cells in light zone (GC B cells in LZ), plasma cells, and memory B cells based on classical marker expression (**Figure 6C**). Comparative quantification revealed a specific decrease in marginal zone B cells in HV-infected pigs relative to the N29 group (**Figure 6D**), suggesting early antigen-handling defects in BLNs (31).

Notably, this altered cellular composition was accompanied by a clear functional deficit. No neutralizing activity was detected in sera from HV-infected pigs at 10 dpi. In contrast, sera from N29-infected pigs had detectable neutralization titers ranging from 1:2 to 1:4 (1.0 to 2.0 log_2_) (**Figure 6E**). Given that high-affinity antibody responses depend critically on help from T follicular helper (Tfh) cells within germinal centers (32), we asked whether HV infection disrupted this essential T–B collaboration. Although the difference was not statistically significant, we observed that the proportion of Tfh and GC B cells in the LZ was numerically higher in HV-infected than in N29-infected BLNs (**Figure 6F**). Yet, functional marker analysis revealed a suggested defect in Tfh activation under HV infection. Single-cell transcriptomics indicated that HV infection induced a significant down-regulation of key functional markers in Tfh cells, including *CD28* (33), a critical costimulatory receptor whose engagement on Tfh cells promotes their differentiation, IL-21 secretion and ability to support B cell responses, and *IL-6R* (34), which is essential for IL-6–driven Tfh differentiation and functional maturation (**Figure 6G**).

We further probed Tfh–GC B cell communication using CellPhoneDB analysis, and found that there were broad apparent impairments of Tfh–GC B communication under HV infection (**Figure 6H**). Key signaling pairs such as *CD58–CD2*, *APP–CD74*, and the *ICAM3–integrin αLβ2 complex* were substantially weakened (**Figure 6I**), which may indicate defective adhesion, co-stimulation, and GC positioning signals required for efficient antibody affinity maturation (35–37). In contrast, N29 infection preserved robust Tfh-GC B cell communication, with interaction strength matching or exceeding that of mock controls (**Figures 6H, I**). This preserved cellular crosstalk correlated well with the efficient neutralizing antibody production observed in N29-infected pigs, suggesting that qualitative preservation of Tfh help—rather than mere B cell numbers—may contribute to effective humoral immunity against PRRSV. Together, these results demonstrate that HV infection may disrupt the functional core of the germinal center response by impairing Tfh activation and subsequent Tfh-GC B cell crosstalk. This candidate mechanism could help explain why highly pathogenic HV fails to induce neutralizing antibodies, while N29 may preserve this critical interaction to support effective humoral immunity.

## Discussion

Up to now, PRRSV remains a devastating threat to the global swine industry (38). A critical unresolved question is how PRRSV modulates the adaptive immunity (13). To address this, we focused on the bronchial lymph nodes (BLNs), which serve as the primary lymphoid organ draining the site of PRRSV replication in the lung (39). We employed a comparative approach, analyzing BLNs from pigs infected with either a highly pathogenic strain HV or its attenuated derivative, N29. The attenuation of N29 was achieved through codon-deoptimization, a strategy that not only reduces viral replication but may also alter host immune recognition. Specifically, the increase in CpG/UpA dinucleotide frequency within viral RNA can enhance its sensing by pattern recognition receptors, thereby modulating early innate immune signaling (40, 41). This design enables us to dissect immune mechanisms in a context of high pathogenicity versus protection. Our findings suggest three candidate aspects of PRRSV immune evasion: altered T cell homeostasis, possible impairment of T follicular help, and the induction of an unconventional T cell subset with potential immunomodulatory functions.

The paradoxical accumulation of CTLs in BLNs during HV infection, despite an overall reduction in total T cells, is a novel finding in PRRSV pathogenesis. Our transcriptomic data suggest that this may arise from disrupted migratory programming rather than local expansion. The significant negative enrichment of differentially expressed genes in pathways controlling chemotaxis, cell adhesion, and cell migration implies that HV infection may be associated with dysregulation of the molecular machinery necessary for CTL egress from lymphoid tissues. This retention of effector cells within BLNs likely prevents their recruitment to pulmonary sites of infection, potentially creating a spatial disconnection between antiviral CTLs and viral replication centers. Although increased local proliferation could contribute to CTL accumulation, the proliferation marker TOP2A was not elevated in this subset, making this explanation less likely. Previous PRRSV studies have reported that functional CTL activity is not detected until 49 days post−infection, despite the presence of proliferating memory CTLs as early as 14 days (42). Our finding of CTL accumulation in BLNs at 10 dpi raises the possibility that sequestration contributes to this delayed functional response. Moreover, acute depletion of CD8^+^ T cells has been reported not to exacerbate PRRSV infection (43), which is consistent with the hypothesis that CTLs are not effectively mobilized to infection sites during the early phase. Such compartmentalization of cellular immunity might represent an underappreciated mechanism contributing to the ineffective viral clearance observed during high-pathogenicity PRRSV infection. Longitudinal validation of this phenotype is challenging due to the high mortality of piglets within approximately two weeks after infection with HP-PRRSV. Similar patterns of lymphocyte sequestration have been documented in other viral infections (44). For instance, in HIV infection, high viral loads dysregulate the function of stromal cell-derived factor-1α (SDF-1α)—a crucial chemokine for CD8^+^ T cell migration—leading to impaired trafficking of HIV-specific cytotoxic T cells to viral replication sites (44). This observation further suggests that impaired lymphocyte migration may be a conserved immune evasion strategy employed by pathogens that establish persistent infections. While our transcriptomic data suggest impaired CTL migration, future studies employing *in-vivo* trafficking assays are required to definitively establish this causal relationship.

Our analysis of the humoral immune response suggests that defective antibody high affinity maturation during PRRSV infection may stem from qualitative rather than quantitative defects in T follicular help. Although the abundance of Tfh and GC B cells remained comparable between HV and N29 infections, Tfh cells in HV-infected pigs exhibited significant down-regulations of key functional markers, including *CD28* and *IL-6R*. Other classical Tfh markers (BCL6, CXCR5, ICOS, IL−21, PDCD1) did not differ significantly between HV− and N29−infected pigs. These observations point to a potential qualitative defect, but functional assays (e.g., IL−21 production, B cell co−culture) are required to confirm impaired Tfh help. However, we need to admit that such validation remains challenging in the pig model due to the lack of well−established Tfh−B co−culture systems and limited access to fresh germinal center B cells. If these defects are indeed functional, they could translate to broad disruptions in Tfh-GC B cell communication, as reflected by the specific attenuation of costimulatory and adhesive ligand−receptor pairs. In contrast, the preserved cellular cross-talk in N29-infected pigs aligns with their high-level neutralizing antibody production, potentially suggesting that qualitative aspects of Tfh help—particularly through *CD58-CD2*, *APP-CD74*, and *ICAM3-integrin signaling axes*—may be important for effective antibody responses against PRRSV. Delayed neutralizing antibody production is a hallmark of PRRSV infection (45). Given that Tfh−GC B cell interactions are central to antibody affinity maturation (32), the impaired communication observed in HV−infected pigs offers a candidate mechanism at the level of specific ligand−receptor pairs. This is consistent with findings from other viral infections. Studies have shown that viruses such as Human Immunodeficiency Virus (HIV) and lymphocytic choriomeningitis virus (LCMV) can drive the expansion of Tfh cells, yet this expansion does not enhance productive Tfh–GC B cell crosstalk (46, 47). Instead, it often impairs Tfh cell function and ultimately compromises the development of neutralizing antibody responses. Thus, our findings identify specific cellular communication networks that are transcriptionally altered during pathogenic infection, and these data extend previous observations of delayed neutralizing antibody production during PRRSV infection and provide hypotheses for future functional testing.

The most unexpected finding is a RAG1^+^ DP T cell subset associated with PRRSV infection. Peripheral re-expression of RAG1 is not entirely unprecedented. In mouse mature B cells, for instance, antigen stimulation in germinal centers induces RAG1, potentially contributing to secondary immunoglobulin gene rearrangements and antibody affinity maturation (48–50). However, evidence for such re-expression in T cells remains scarce. To date, RAG1 expression in peripheral T cells has only been reported in the synovial tissue of patients with rheumatoid arthritis, where authors hypothesize a possible “extrathymic T cell development” process, though direct evidence confirming RAG1 expression in T cells remains lacking (51). Here, identification of a RAG1^+^ DP T cell subset in PRRSV-infected BLNs offers a rare example of virus-associated peripheral RAG re-expression in the T lineage. The recapitulation of a thymic developmental signature in peripheral lymphoid tissue suggests that PRRSV may trigger aberrant differentiation or developmental reprogramming. Notably, pigs inherently possess a large population of conventional peripheral CD4^+^ CD8^+^ double−positive T cells, which are generally regarded as memory cells originating from activated CD4^+^ T cells (52). Previous work has also confirmed that PRRSV infection elevates the frequency of the conventional DP T cells in lymphoid tissues (53, 54). However, the RAG1^+^ DP T subset identified in our study is distinct from these conventional DP T cells, as it expresses V(D)J recombination machinery and lacks typical memory markers. The transcriptional profile of this subset, with co-expression of V(D)J recombination machinery and lipid antigen presentation molecules, suggests its dual functionality—potentially enabling both TCR revision and unconventional antigen recognition. Due to the lack of validated commercial antibodies against porcine RAG1, protein-level confirmation of this subset could not be performed at this time point. Therefore, its existence and proposed functions remain to be verified in the future. To test these functional hypotheses, future studies should combine fluorescence-activated cell sorting (FACS) of this subset with *in vitro* antigen stimulation assays. Specifically, sorted RAG1^+^ DP T cells could be exposed to lipid antigen-presenting cells or CD1-restricted antigen panels to directly evaluate their lipid antigen recognition capacity. Parallel TCR sequencing before and after stimulation would clarify whether these cells indeed undergo active receptor recombination in the periphery, though this technique is still technically immature for porcine samples. Such experiments are essential to distinguish whether this subset functions in antigen-driven immunity, TCR repertoire diversification, or viral-mediated immune diversion. Its preferential induction by the attenuated N29 strain further suggests that it represents a beneficial immune adaptation rather than a purely pathological phenomenon. These possibilities warrant direct experimental testing and will significantly advance our understanding of PRRSV immunobiology and peripheral T cell plasticity.

Several limitations of our study should be acknowledged. While single-cell RNA sequencing provides unprecedented resolution of cellular states, it primarily generates hypotheses rather than establishing causation. The proposed migratory defect in CTLs requires confirmation through direct trafficking experiments, which remain challenging due to the lack of validated pig−specific antibodies now. Similarly, the functional significance of the identified RAG1^+^ DP T cell subset requires validation through isolation and functional assays. Furthermore, this RAG1^+^ DP population is not detected in all PRRSV-infected samples, suggesting it may be transient. Since we collected samples at a single time point and used a limited number of biological replicates, additional serial sampling and validation with larger independent cohorts are therefore needed to fully characterize the dynamic immune responses and enhance the generalizability of our conclusions.

In conclusion, our study provides a high-resolution view of how PRRSV reshapes the BLN immune microenvironment to evade host immunity. By systematically comparing pathogenic and attenuated infections, we have highlighted specific candidate immune evasion strategies for PRRSV. These findings not only advance our fundamental understanding of PRRSV pathogenesis, but also point to specific cellular interaction networks that could be targeted for therapeutic intervention or vaccine improvement.

## Data Availability

The raw single-cell RNA sequencing data reported in this paper have been submitted to China National Center for Bioinformation GSA database (http://ngdc.cncb.ac.cn/gsa/), with accession number of CRA045821.
